# The Perception of Novel Consonant Clusters: A Comparison of Salience and Sonority

**DOI:** 10.3390/brainsci16060583

**Published:** 2026-05-29

**Authors:** Marina Oganyan, Matthew C. Kelley, Yuan Chai, Akira Omaki, Richard A. Wright

**Affiliations:** 1 Department of Linguistics, University of Washington, Seattle, WA 98195, USA; marina0@uw.edu (M.O.); yuc521@ucsd.edu (Y.C.);; 2 Linguistics Program, Department of English, George Mason University, Fairfax, VA 22030, USA; mkelle21@gmu.edu

**Keywords:** sonority, perception, phonology, salience

## Abstract

**Highlights:**

**What is the main finding?**

- We find that salience is a better predictor than sonority of consonant cluster simplification during real-time perception.

**What is the implication of the main finding?**

- Our main result suggests that consonant cluster simplification in perception can be explained using principles of acoustics and human audition.

**Abstract:**

**Background/Objectives:** In human language, speech sound units, referred to as segments, are rigidly ordered. Certain orderings are typologically common, while others are typologically rare. This pattern has led linguists to posit a scalar segment-intrinsic feature, referred to as “sonority,” and a hierarchy relating to ordering referred to as the “sonority hierarchy.” In generative phonology, it has been proposed that the sonority hierarchy is present in the innate underlying grammar as the Sonority Sequencing Principle (SSP) and variations thereof. In grammar-based approaches it is common for speech sounds to be abstractly classified based on manner with obstruents being the least sonorous and vowels being most sonorous and with three levels of adherence to the SSP: 1 strict adherence with a sonority rise as in the sequence /pla/, 2 slight adherence/slight violation with a sonority plateau as in /pta/ or /mna/, and 3 violation with a sonority fall before the vowel as in /lpa/. Some linguists have proposed that segmental ordering is the result of generalized pressures relating to speech perception, cognitive processing, and gestural coordination, and therefore not part of the underlying grammar. This study examines the relative contribution of perceptual salience (defined as loudness and segmental recoverability) to segmental ordering. **Methods:** We used eye-tracking in the visual world paradigm to track real-time processing of perception of novel consonant clusters. We compared whether salience or sonority had a stronger association with how participants simplify clusters in perception, where salience and sonority had different predictions. **Results:** We found that participants looked more towards salience-predicted competitors than sonority-predicted ones prior to focusing on the target. **Conclusions:** This finding is in line with theories based on acoustic and auditory salience being the strongest predictors of perceptual ease.

## 1. Introduction

**Figure 1:**
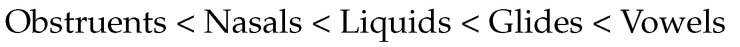
Sonority ordering from least sonorous to most sonorous as in Clements [3].

In human language, individual speech sounds (commonly known as segments) cannot be arbitrarily ordered but must be organized based on rigid restrictions, known as phonotactic rules/constraints. Moreover, as discussed thoroughly in the literature [1,2,3,4,5], there are clear typological patterns across languages in how segments are ordered. When sequences of consonants occur in the same syllable, the most commonly observed pattern is an obstruent (such as /b/) followed by a liquid (such as /l/) or glide (such as /w/) followed by a vowel (such as /a/) and closing in the opposite order, as in the syllable in the English word “block” /blak/. The reversed consonant order, /lba/, is extremely rare in languages, and there is an implicational relationship between the two orderings such that if a language allows a sequence like /lba/ it also allows a sequence like /bla/ but not the other way around. This pattern has led to two proposals. The first is that there is a scalar segment-intrinsic feature, referred to as sonority, which can be correlated to intensity or perceived loudness, and which results in a sonority hierarchy [4,5]. Although there are many variants of the sonority hierarchy, some of which can be quite intricate, as discussed thoroughly in Parker’s work [4,5,6], a common version is illustrated in Figure 1.

The second proposal is that languages order sounds based on their sonority with the lowest sonority at the margins of a syllable and the peak of sonority at the middle, formalized as the Sonority Sequencing Principle or SSP [3]. Of the many other variants of the SSP, the most phonetically grounded is Parker’s Sound Level Protrusion, which posits that sonority sequencing creates intensity modulation with peak intensity at the syllable nucleus [4,5].

While the notion of sonority and the sonority sequencing principle have their roots in linguistic theory, the concepts have had a profound impact on research in cognitive scientific research and neuropsychological and neuroanatomical studies on language acquisition and processing [1,7,8,9,10,11,12]. Because it is often invoked as an explanatory principle in cognitive and neuropsychological studies, understanding underlying forces at work in sonority is both relevant and important to fields in neuroscience.

The earliest uses of the sonority hierarchy and sonority-based ordering were purely descriptive [13]. However, in the latter half of the 20th century, variants of the SSP were proposed by generative linguists to be part of Universal Grammar (UG) and, therefore, part of the innate grammatical knowledge of language structure that all humans possess at birth regardless of their matrix language [1,3,14,15]. On the other hand, some linguists have explored the possibility that the patterns described in the SSP and related work result from domain-general restrictions on motor coordination and auditory perception [16,17,18,19,20].

In a series of experiments, Berent and colleagues tested whether a version of the SSP predicted behavioral responses to novel onset clusters in adults and children [2,21,22,23,24]. They found that, in production, the greater the sonority violation, the more likely epenthetic vowel insertion is. This is interpreted as a repair strategy for an ill-formed cluster, and it is commonly attested across languages and documented in the phonological literature. One study on adult silent reading found that ill-formed clusters with larger sonority violations took longer to distinguish from their well-formed disyllabic counterparts (e.g., lbif–lebif) [2]. This contrasts with the better-formed, but still unattested, clusters (e.g., bnif–benif). The reading latency effect was robust to tests for lexical frequency. They interpret the results to indicate that the ill-formed clusters undergo a cognitive repair process through vowel insertion even when the utterance is not read aloud.

In a follow-up study from Berent and colleagues with 4-year-old children [24], clusters which are well-formed in Russian but ill-formed in English were presented auditorily as names of visual stimuli. The clusters ranged from sonority reversals (e.g., lbif) to sonority plateaus (e.g., bdif) to sonority falls (e.g., bwif), all of which were unattested in English. The children were presented with an initial auditory–visual stimulus, with or without an epenthetic vowel followed by a second auditory–visual stimulus with the counterpart syllable structure, and they judged how good a match the second auditory stimulus was to the first. For example, a child might hear lbif as the name of a visual stimulus, then hear lebif and give a goodness-of-match judgment. They found that children were least likely to notice the epenthetic vowel distortion in sonority reversals, followed by plateaus, and children were most likely to notice the distortion in sonority falls. They interpret their results as consistent with previous findings that children’s grammatical knowledge, like adults’, mirrors sonority restrictions and can therefore be interpreted as evidence for a sonority component in the children’s UG. In their conclusion, Berent et al. [24] acknowledge that there remain several unexplored areas about the nature of sonority and segmental sequencing that are not tested or explained by their findings. One area that is worth exploring is whether some of the perceptually motivated phonotactic constraints relating to salience and recoverability [19,20] might play a role in what a listener notices in an experimental stimulus or when they encounter a novel consonant cluster in a new language. While this question does not immediately address the theoretical question of whether the subject is relying on UG in noticing variations in novel sequences, it is a valid question to ask why a subject might notice variation in one sequence and not in another.

For the purposes of the present study, salience is defined as the likelihood that a listener notices that an event has occurred. It is dependent on two dimensions of sounds and sound sequences: loudness and contrast. Loudness is correlated with a segment’s internal intensity and aligns with Parker’s definition of sonority [4,5]. For example, the fricatives /s/ and /f/ are both voiceless fricatives and, from an abstract point of view, have equal sonority. However, from Parker’s intensity-based definition of sonority, they are far apart on a sonority scale with /s/ having a much higher intensity and, therefore, salience than /f/. Contrast relates to how perceptually different two sounds are. While the sequence of two fricatives constitutes a sonority plateau from an abstract point of view, the sequence /sf/ is more salient than the sequence /s∫/ because the spectra and intensity of /sf/ are more divergent (i.e., more contrastive) than /s∫/.

Recoverability is related to the probability that the place, manner, voicing, and even the presence of a segment can be recovered by a listener in a particular environment. It is related to salience, but it is also tied to how cue-encoding in strings of sounds is modulated by the coordination of articulatory gestures. For example, in the sonority plateau /pt/, if the gestures are timed such that there is a release burst for both the first and second sounds, conveying information about the place, manner, and voicing, the sounds are recoverable. However, if the gestures for /p/ and /t/ overlap such that the release burst for the /p/ is subsumed by the closure of the /t/, only the information for /t/ is preserved. Notice that for other sonority plateaus, pulling the gestures apart will not increase recoverability, such as in the cluster /mn/ since nasals do not build up intraoral air pressure and therefore do not produce a release burst. If the second sound’s spectrum is shaped by the first sound, as in the sequence of a stop followed by a glide as in /bwa/, critical information about the first sound /b/ is carried by the release burst and by frequency modulations in the second sound /w/ and is, therefore, recoverable even when the two gestures overlap.

To test the role of salience and recoverability in whether a listener notices and registers the sounds in a sequence of consonants, the current experiment uses the stimulus sequences from Berent and colleagues, recorded by a professionally-trained phonetician, and curated by a native speaker of Russian, to create stimuli which are paired with visual stimuli. In this paper we refer to salience and recoverability using just the simpler term “salience.”

### The Present Paper

Our paper aims to compare how much explanation of cluster perception is afforded by salience versus sonority. To make this comparison, we investigated the perception of novel (to English) consonant clusters predicted to be of varying salience- and sonority-goodness. To do so, we used eye-tracking in a visual-world paradigm. This technique allows for real-time comparison of confusions in perception. We were testing whether or not participants respond to the presented cluster more in line with salience or sonority’s predictions. Additionally, we sought to replicate the basic cohort competitor effect [25,26], where initial gaze is split between the target and competitors with the same onset before enough of the word has been perceived to disambiguate between the two. This serves as the premise for the main research question in this study.

Our main research question was whether salience or sonority was a better predictor of participant confusions during perception. We keep in mind that salience has greater correspondence with the material substance experienced during speech perception—the acoustic and auditory properties of speech—than sonority. From this point, our prediction is about earlier processing times during perception. Namely, during that earlier time, we believed that participants would look more often to the competitor that is easier to perceive according to salience. A secondary post-hoc research question was about what our scales for salience and sonority meant and how well they predicted relative looks to target.

## 2. Experimental Design

### 2.1. Visual Stimuli

For visual stimuli in our visual-world paradigm, we used novel creatures from Van de Vijver and Baer-Henney [27], with permission. We used ratings obtained on the Amazon Mechanical Turk platform to verify that there was no implicit bias for or against the associations between the novel creatures and objects and their names. None of the novel creatures or objects showed any above-chance association with their names.

### 2.2. Materials

The clusters we used were grouped relatively evenly into having the best, medium, or worst salience or sonority. These groups are shown in Table 1.

For salience, the clusters with the best salience were those that were expected to show high perceptibility based on acoustic and auditory principles. The medium salience clusters were those where it would be challenging but reasonable to hear both segments in the cluster. The worst salience clusters were those where it was expected that a participant might only hear one of the segments in the cluster. For sonority, the best clusters had a rise in sonority, the medium clusters had equal sonority, and the worst clusters had a fall in sonority.

In addition, predictions were made on recoverability of each consonant within a cluster based on salience and sonority. The cases where these differ serve as the basis for analysis 1. For salience, the sound that had most information present for its recoverability was selected. For sonority, the sound with the lowest sonority was selected to result in the greatest sonority difference between the consonant and vowel. For example, for the clusters /vd/ and /dv/, salience predicts good recoverability for /v/ in both clusters but poor recoverability for /d/ when it precedes /v/ instead of a vowel. Fricatives have internal cues to their voicing, manner, and place and are therefore less reliant on a neighboring vowel or strict gestural coordination for recoverability. Moreover, the /v/ frication is salient and alerts the listener to its presence. On the other hand, stops have no internal cues at their peaks of stricture and are therefore reliant on their release bursts and neighboring vowels for cue recoverability. The release burst of /d/ has low intensity, as is the case for all voiced stops [28], and is easily confused with the overlapping frication noise of the following fricative. In the cluster /tf/, /t/ is ranked more recoverable because the release burst is high in intensity, as is typical of in voiceless stops. However, since sonority predicts rising sonority values in an onset cluster, /dv/ adheres better to the SSP than /vd/ does.

It is important to note that the salience scale, in particular, is a discretized representation of a gradient property of clusters. The categories here are proffered instrumentally, but we do not posit them to be categories literally represented in the brain/mind. On the other hand, the sonority scale is tied to three discrete patterns in sonority sequencing, those of increase, plateau, and fall within the onset.

#### 2.2.1. Critical Trial Stimulus Design

One creature and one object word were created for each cluster for a total of 32 non-filler stimuli. For a given cluster, names were chosen starting with the cluster, followed by the vowel in common with the competitors’ first syllable, and then choosing a unique end to the name. One item was selected that began with the first sound in the cluster, and another item was selected that began with the second sound in the cluster. By means of an example, one trial consisted of the creature [m(ə)nʌvɚ], the object [m(ə)nʌpn̩], the distractor *mushrooms* [mʌ∫ɹumz], and the distractor *nuts* [nʌts]. The full list of the stimuli for each trial can be found in Appendix A.

Two versions of each trial were created, one where the creature had an epenthetic schwa breaking up the cluster, and another where the object had the epenthetic schwa. Within the trial, these versions were kept consistent.

#### 2.2.2. Filler Trial Stimulus Design

**Figure 2:**
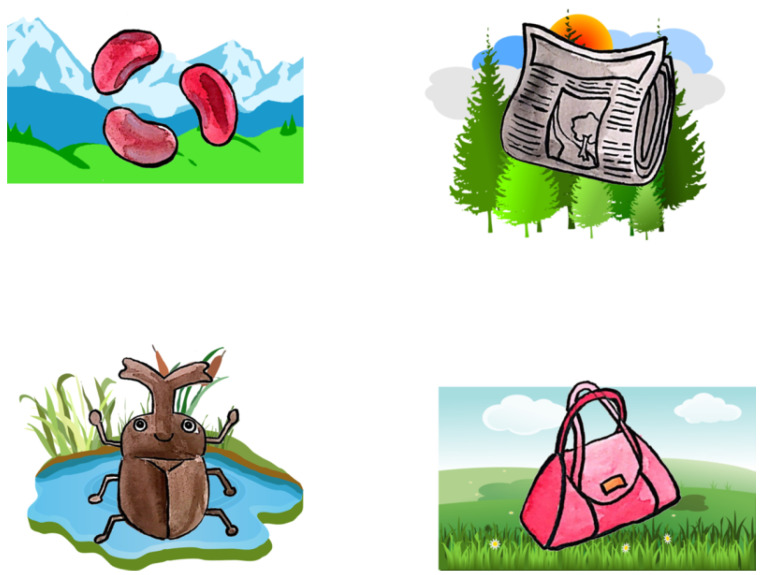
Example of a trial. The quadrants show *beans* in the mountains in the upper left, *beetle* in the pond in the lower left, *newspaper* in the forest in the upper right, and *purse* in the field in the lower right.

The fillers were all real words, with a trial consisting of a target, a cohort competitor, and two unrelated words. The cohort competitor had the same two initial sounds as the target: a consonant followed by a vowel. The other two words had no overlap with the target in initial sounds. See Figure 2 for an example.

#### 2.2.3. Stimulus Recording and Preparation

The stimuli were recorded on a Zoom H4n recorder in a sound-attenuated booth at the University of Washington by the last author. For the trials with the novel clusters, two different versions of each scene were recorded. In one version, the creature had the cluster and the object had the cluster broken up with an epenthetic schwa. In the other version, it was the object that had the true cluster and the creature that had the cluster broken up by an epenthetic schwa. The speaker paused before and after each stimulus to ensure that there were no effects of coarticulation to confound the natural perception of the clusters. The pauses were subsequently shortened to sound natural when listened to.

We manually reviewed each recording, using auditory judgment and examination of both the spectrogram and waveform to determine that the clusters were pronounced with no epenthetic or excrescent vowels. We also ensured that each cluster that was supposed to be broken up with an epenthetic schwa was indeed broken up. When a creature or object name was determined to be produced incorrectly, the sentence was re-recorded, or, in some cases, if there was a previous recording that had the correct production, the recordings were spliced together to replace the incorrect one. When performed, the splicing was made to happen at zero-crossings that preserved the positive- or negative-going trend of the waveform. The result of the splicing was inspected auditorily and spectrographically to ensure that no pops from discontinuity occurred. The auditory judgment also ensured that the prosody matched. If splicing was attempted but could not be resolved satisfactorily, the sentence was instead re-recorded.

### 2.3. Participants

Participants were recruited through posters hung around the University of Washington Seattle campus advertising the study. The first 25 participants emailed the study team, and a member of the study team discussed the participant’s eligibility based on language background and possible vision or hearing impairments. Subsequent potential participants completed a screening questionnaire by scanning the QR code on the flyer, providing information on vision and hearing status as well as basic language background. The study team then contacted eligible participants and invited them to participate in the experiment. Included participants reported normal or corrected-to-normal vision sufficient to read the computer display. No included participants reported hearing loss/impairments. Those requiring correction wore contact lenses, and if this was not possible, the participant was not included.

While the results of a power analysis would help determine the sample size that we need, the process for doing so is not clear. We employed mixed-effects models for most of our analyses, which require the use of simulations to produce a power analysis. There exist processes for doing so for (generalized) linear mixed-effects models [29]. However, we are not aware of any such process for the generalized additive mixed models (GAMMs) [30] that we employed to model the nonlinear trends in our data. For this reason, we opted instead to recruit as many participants as we reasonably could. This choice was informed by the number of participants in previous experiments comparing fixation curves between different competitors, such as Porretta and Kyröläinen [31] and Kalénine et al. [32], which had 50 and 17 participants, respectively.

Eighty-three participants participated in the experiment, and 18 were subsequently excluded due to hardware/software malfunction, unanticipated distracting noise coming from elsewhere in the building, or for later disclosing that they did not speak English before the age of five. One participant that was run was later excluded on the basis of speaking Hindi, which permits the cluster [d̪v] as in [d̪vip] ‘island’ [33], which is close to the [dv] cluster in our stimuli. Another participant revealed that they spoke Russian, which contains all of our clusters, so that participant was excluded as well. Other exclusions were made due to an error in data collection, such as not recording fixations, cables coming loose, or loud background noise distracting the participant.

Ultimately, 65 participants’ data were kept (of those who reported demographic information, there were 43 women, 12 men, 1 transgender man, and 3 non-binary participants; mean age = 22 years). We have language data for 40 of the included 65 participants. Of those 40, four spoke (an)other language(s) before the age of five. In addition to English, one participant also spoke Cantonese and Vietnamese, while other the three spoke one of Korean, Chinese (variety unreported), and Spanish. None of the languages spoken by the included participants had the onset consonant clusters examined in this study, reducing the influence of prior linguistic experience on the results. Participants were assigned to one of four stimulus lists (List A = 16; List B = 17; List C = 16; List D = 16).

There is literature suggesting that multilingual individuals have an advantage in phonetic learning [34,35,36,37]. However, as elaborated on in Section 5, we did not observe a significant difference in looks to target between mono- and multilingual listeners in critical trials.

### 2.4. Procedure

Before the experiment started, participants received standardized instructions that framed the task as helping a fictional character, Uncle Ron, find things. Stimulus presentation and data collection were programmed in Experiment Builder (SR Research, Ottawa, ON, Canada). Each participant completed 16 experimental trials. In each trial, a short animation introduced four objects. The image of Uncle Ron then appeared at screen center; participants fixated on Uncle Ron to complete central fixation before the trial sequence continued. The animation then placed each object into one of four equal-sized scene backgrounds (forest, field, pond, or mountain), displayed in the four corners of the screen. The participants were asked to identify the location of one of the four objects introduced earlier (e.g., “Can you help Uncle Ron find the puppy?”). Participants indicated which scene contained the target by giving a verbal response (e.g., “forest,” “field,” “pond,” or “mountain”). Audio prompts introducing the objects and the tasks were played over a speaker. The experimenter recorded each response both on paper and on a computer by pressing a key on the keyboard corresponding to the screen position. The session ended after all 16 trials in the participant’s assigned stimulus list were completed.

The eye movements of the participants were recorded throughout the experiment with a desktop-mounted EyeLink 1000 Plus (SR Research, Ottawa, ON, Canada). The eye tracker was configured for monocular tracking of the left eye for 43 participants, and 22 had their right eye tracked. Participants sat with their heads unrestrained and watched a screen that displayed visual stimuli. The camera’s height and angle were adjusted to output robust tracking signals within recommended ranges (pupil threshold 75–110; corneal reflection threshold < 230). Before the experiment, we conducted a five-point calibration and validation (targets at the top, bottom, left, right, and center of the screen). Calibration established the mapping from the raw eye camera signal to screen coordinates; validation then assessed the accuracy of that mapping by comparing computed fixation positions to the known target locations. Calibration/validation cycles were repeated, with camera adjustments as needed, until the difference at every validation point was smaller than one degree of visual angle. The experimental trials began after the calibration and validation were completed.

### 2.5. General Data Preparation

The data were processed using VWPre [38]. The time was offset by 200 ms to help visually center the effects after the participant heard the target item. A window ranging from −200 ms to 1200 ms was used for analysis and plotting, though the region we were most interested in was from approximately 200 ms to 400 ms, representing a reasonable amount of time after hearing the stimulus for the participant to begin fixating on the target. More details on data preparation are given when relevant in the upcoming sections.

## 3. Replication: Filler Analysis

In our filler analysis we compared looks to the target versus cohort competitor over time. We predicted that participant gaze would focus on both of these equally before enough of the word was perceived to distinguish between the two and focus moved to the target.

### 3.1. Methods

We performed a cluster-based permutation analysis (CPA) to identify the time course in which looks to target significantly differed from those to the competitor [39,40,41,42].

### 3.2. Results

**Figure 3:**
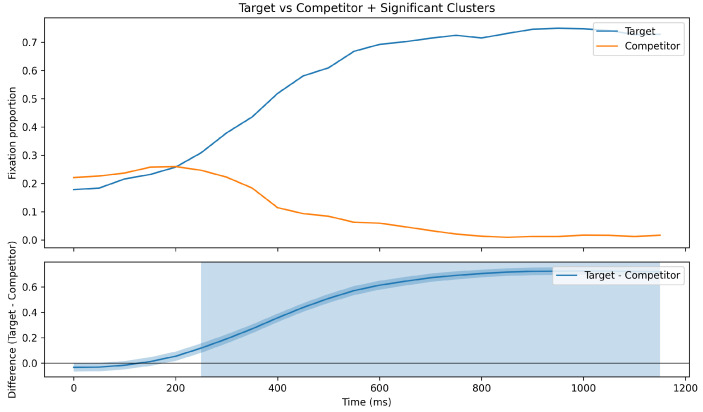
(**Top**) Proportion of fixations at target vs. cohort competitor. (**Bottom**) Difference between target and cohort; shading indicates significant difference.

As seen in Figure 3, a significant cluster was observed between 250–1150 ms (*p* < 0.001). No significant differences prior to 250 ms and visually observe near overlap prior to that point. This suggests that, initially, participants looked at both the target and cohort competitor before focusing once more on the target word.

## 4. Analysis 1

In the first analysis of our critical trial data, we compared fixations to the two competitor conditions, sonority-favored and salience-favored, after stimulus presentation. Higher percent of fixation to the either of the conditions prior to disambiguation suggests higher confusion rate, i.e., that the participant confuses that condition more with the cluster of the target.

### 4.1. Methods

To analyze the data for significance, we used GAMMs. GAMMs allow for the specification of nonlinear effects without needing to manually perform feature engineering as might happen with using orthogonal polynomials as in growth curve analysis [43].

When a nonlinear smooth effect is fit in a GAMM, a smoothness penalty is applied to it, preventing unjustified nonlinearity and reducing the propensity to overfit. Because of this behavior, GAMMs can reasonably handle fine time sampling of the fixations. Autocorrelations can also be handled in the model structure [44], which can otherwise be a problem for estimating the certainty of the effects in a time-series model [44,45].

The data were processed so that each sample from the eye tracker represented four rows, where each row represented the fixations to a given quadrant on the screen, as in previous work [32,43]. This allowed for the response variable to be whether a participant looked or not.

The predictor variables for the main model then included a fixed parametric effect for the item the fixations were toward, a smooth effect for time, and a smooth effect for time by which item the participant was looking at (thereby fitting different curves for looks to each item for a trial). We also fit a random smooth for time by event, which handles random effects for both speaker and item while also accounting for autocorrelation [44]. The model used a binomial family for the regression.

### 4.2. Results

**Figure 4:**
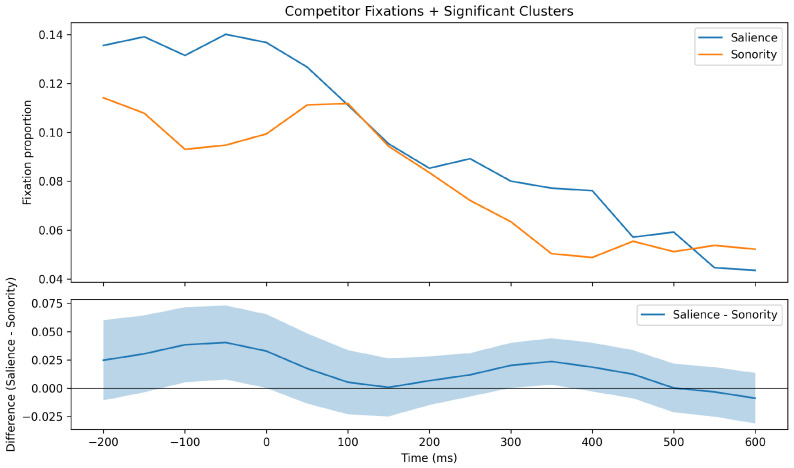
(**Top**) Proportion of looks to salience vs. sonority competitor. (**Bottom**) Difference in proportion of looks.

A visual representation of the proportion of looks to the salience and sonority competitors can be seen in Figure 4. Using a difference analysis from the the GAMM calculated with itsadug [46], a significant region of difference was found between −200 ms to 40 ms, and another between 238 ms and 280 ms. In both of these regions, the competitor predicted to be looked at by salience was looked at more often than the competitor predicted to be looked at by sonority. The table of coefficients for the GAMM can be seen in Table A2 in Appendix B.

## 5. Analysis 2

In this analysis, we focused on the secondary research question of whether salience or sonority scores of clusters were meaningfully associated with looks to the target. It was a planned post-hoc analysis we undertook to better understand the behavior of certain types of clusters on participant behavior. The results of the present analysis do detract from the main finding of the previous analysis that when sonority and salience differ on predicting how a novel cluster will be simplified in perception, salience provides a better match to participant behavior.

### 5.1. Methods

The data used in this analysis were the same as the data from the previous analysis. The modeling question was different, however. Rather than examining how different properties of the stimuli affected looks to competitors, we examined how the different sonority and salience levels affected looks to the target. In this analysis, we used an autoregressive (AR) model to account for autocorrelation since the random smooths for time-by-event resulted in nonconvergence.

We fitted a preliminary model to check for an influence of language background on responses. Language background was treatment-coded as a binary factor with monolingual as the reference level and the other level being multilingual. A random smooth over time was fit for participants with the factor-smooth basis and the *m* parameter set to 1. The predictors also included a parametric effect for language background and a time-by-language-background smooth interaction fit, as well as a smooth for time. The models were fit using a binomial distribution as the family, resulting in a logistic-style regression. The “successes” in each observation were coded as whether the participants were looking at the target or not.

Subsequently, we fitted two different models using our salience and sonority scores. The basic model structure had a global smooth effect for time. As in the language background model, a random smooth over time was fit for participants with the factor-smooth basis and the *m* parameter set to 1. The salience model then had a parametric effect for salience level and a time-by-salience-level smooth interaction fit. The sonority model had an analogous structure using the sonority variables in place of the salience variables. As in the language background model, the models were fitted using a binomial distribution as the family. The “successes” were coded the same. AR models for logistic-family GAMMs are allowed when the discrete parameter is set to a value of TRUE, despite previous claims in the literature [38,41]. To find the rho parameter value for the AR model, we used the procedure described in van Rij et al. [44] via the start_value_rho function from itsadug. The autocorrelation in the model residuals decreased drastically after specifying the AR(1) structure, so we interpreted the GAMMs with the AR(1) models as our final models.

### 5.2. Results and Discussion

The model for language background did not show a significant effect for the parametric term for language background (β=0.001, SE=0.062, z=0.020, p=0.984). A difference plot comparing the looks to target over time also did not return any differences for the time-by-language-background smooth interaction. It may well be the case that the random smooth for participants is already handling this kind of by-participant information, leading to the language background information being redundant. Due to not having significant effects of interest, we do not believe language background had an effect on our results and do not interpret this model further.

The coefficients for the GAMMs for the salience and sonority predictors are given in Table A3 and Table A4, respectively, in Appendix B. Note that the *p*-values reported do not directly indicated where there is a time-course difference between predictors, which is instead shown in the upcoming difference plot results. The models are largely comparable in interpretation, that the two different predictors have similar effects on the looks to target.

**Figure 5:**
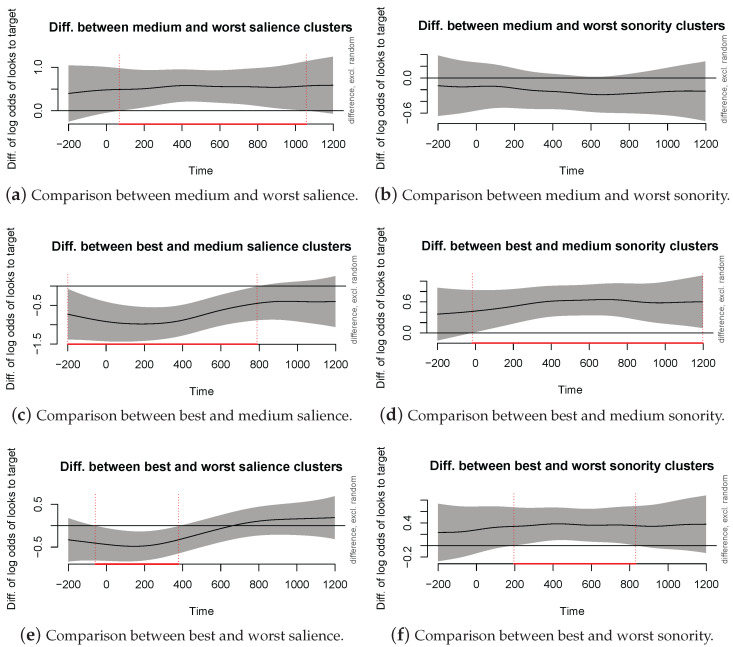
Difference plots between the different categories within the salience and sonority variables. For each comparison, the difference shown is the first level minus the second level. The regions where the difference is statistically significant are indicated by the red demarcating line at the bottom. Positive values indicate looking at the target more often for the first level than the second, while negative values indicate looking at the target more often for the second level more than the first.

Where the differences emerge between the salience and sonority models is when the levels of the time-by-factor interactions are compared. Calculated using the itsadug package [46], smooth difference plots separately comparing each level within the sonority and salience plots are given in Figure 5.

The way we interpret these plots is that only our salience score was able to pick out a coherent set of clusters, those with medium value. The medium salience clusters are different from the worst clusters in that they are looked to more often as time progresses. We interpret this to mean that it was ultimately easier for the participants to fixate on the target for the medium salience clusters as compared to the worst ones. In another trend, the best salience clusters are different from the medium clusters in that they are looked to less often as time progresses. This trend may mean that participants found the target more quickly when the cluster had the best salience score as opposed to a medium salience score and thus had time to look elsewhere. Or, it could be that there was something inherently challenging about some of the clusters in some of the salience groupings that did not get reflected in our ranking. It is also possible that the best clusters caused participants to look at the item with the cluster broken up by epenthesis since they heard both sounds, thereby leading to fewer looks to target for the best clusters.

It is worth keeping in mind that there were only three clusters given a medium score: [dl], [gd], and [tf]. It is possible that having more clusters in this category would help better contour the effect. It may be the case that there is some kind of processing advantage for medium salience, perhaps related to signal smoothness [47,48] by providing less “peaky” information content despite lower audibility and recoverability. A greater diversity of clusters of this type may illuminate this pattern further.

Contrarily, there was no detected difference when comparing the worst sonority clusters with the medium sonority clusters. We do not interpret the lack of an effect as evidence of no effect, however. It may be the case that a greater sample of clusters (and therefore participants and/or trials) is needed to be able to detect the difference, though the numerical trend suggests that the medium sonority clusters resulted in fewer looks to target than the worst. There is a straightforward trend of the best sonority clusters resulting in more looks to target than the medium- and worst-scored clusters. There would be a synergy between this approach for salience and more continuous treatments of speech phenomena [49,50,51,52]. It is also worth considering that salience, as a continuous property itself, should be treated more continuously within an experiment.

Overall, the results we have here are suggestive of trends. However, a more full investigation into the relative rankings of clusters would require more exhaustive sampling of both cluster types and participants, especially of different language and demographic backgrounds. Still, it is interesting that both scales seemed to isolate different aspects of participant behavior.

## 6. General Discussion and Conclusions

Much as predicted, salience was more strongly associated with participant behavior in our primary analysis. We do not wish to cast aspersions on sonority per se. Rather, we believe, as do Henke et al. [17], that sonority sequencing represents an optimization of salience in the structuring of speech patterns. This conclusion accords well with salience being a better predictor of behavior than sonority with our stimuli that do not follow the sonority sequencing pattern (as borne out in the greater proportions of fixations to the salience-predicted competitor). Note, however, that the predictions and patterns of the sonority hierarchy and sonority sequencing principle are derived from the data they are describing. Sonority, then, is a statement of the patterns it identifies. Salience, on the other hand, coming from more external factors like human audition and the structure of physical signals, can explain why speech patterns in the way that it does. Under this light, it is clear why sonority has less explanatory power in the statistical models since sonority itself is not an explanation but a description.

We also note that the effects we observed here were for a relatively small sample (when looking at the total number of trials) over a short span of time, taking place in a controlled lab setting. We believe that the effects of salience would be even more pronounced in a less controlled and more difficult listening environment where ambient noise may contribute to masking some auditory properties of the speech signal [53,54]. While it seems like an obvious point that some auditory properties become less salient when there is noise that may mask them, this is exactly the reason we believe that salience itself is worth appealing to when discussing consonant cluster sequencing. In other words, as a notion, salience directly relates to physical aspects of the produced speech signal and the listening environment, which explains both why some clusters would be avoided in speech and why some clusters may be more vulnerable to change than others.

Sonority sequencing does not address this aspect of the variable audibility of consonant clusters short of making ad-hoc categories based on subsequences that may not have much internal coherence. As an example of the latter, sonority sequencing cannot distinguish between [st] and one of the clusters we tested like [ft] without introducing more ranks. Salience and cue robustness straightforwardly account for [st] being more audible by virtue of the flanking [s] having sibilant energy that increases its audibility to make up for the absence of a flanking vowel, a property which [f] does not have.

It is worth admitting that salience is harder to operationalize into a formal system than the rank-ordering of the sonority sequencing principle. One possible solution would be to continue work that interfaces sonority with salience, finding ways to describe sonority sequencing through appeals to salience. In this way, previous work that involves sonority sequencing need not be thrown out with the bath water, so to speak, but previous and future work can be reconceptualized in a more grounded way that affords a more basic and thorough explanation than would rank-order patterning. The physical backing of sonority would then be situated with the communicative goals of speech in mind, a more holistic and functional backing than relative loudness. This resituation has been suggested previously [17], and we proffer it again as a perceptual and auditory grounding for any constraints on consonants that have involved sonority.

Without fitting the sonority hierarchy into the broader landscape of salience, much of the acoustic–perceptual space that exists for consonant clusters is underexplored. Focusing so heavily on only the options for clusters that maximize perceptibility leaves a blind spot for all speech researchers. What might be said about why a cluster that has not been optimized as such exists in a language? Appealing only to constraint rankings as in some previous research [15,24,55] leaves unanswered the functional aspects of why clusters take the form that they do.

In terms of implications, we believe that our results speak to a need to incorporate more physically grounded concepts like salience into linguistic and phonological theory. Insofar as theory in linguistics is about modeling and explaining human behavior, it is important to have models that relate to embodied experience with the material substance of language, in the case of oral languages, the acoustic speech signal. The connection between this material substance and other areas of inquiry involving language, such as neurolinguistic studies, should not be understated. As we discussed in our introduction, study designs outside of linguistics use linguistic constructs and concepts, and the closer the relationship there can be between them and other measured quanta like brain activity, the better.

There is also something to be appreciated in terms of parsimony about behavior being related to physical properties outside the mind/brain. In this specific case, relying on sonority requires an assumption that there is a literal representation of rank-ordering of sounds in the mind that the brain must somehow implement or otherwise be sensitive to. Meanwhile, using salience allows acoustic properties of the speech signal and established properties of human audition to explain behavior in cluster simplification. While it could be argued that sonority could be indexing some physical quantity, such an approach renders it an ersatz salience. In sum, salience affords greater ability to explain a wider amount of human behavior at a lower complexity than would sonority.

## 7. Limitations and Future Directions

This study had limitations when it came to comparing different levels of salience violations due to the stimuli being largely designed for sonority-based comparisons. In future work, it would be important to extend this study with stimuli specifically selecting for balanced and varied differences in salience. This expansion would be important not just for understanding different cluster types but also for developing relative measures of salience. Additionally, it would further tie salience to measurable, physical phenomena as occur in experimental science.

Additionally, regarding participants, running this study with children (as already afforded by the design of this experiment) would allow investigation into the acquisition of clusters in the L1 and what factors are involved in that process. For example, it may be that some clusters are easier at different ages, and the way that this pattern corresponds with salience would pave the way for further linking audition to acquisition processes. It is also possible that asking participants about sensory issues beyond vision and hearing, such as autism spectrum disorder or attention deficit/hyperactivity disorder, would help control for non-linguistic influence on the results (this point was helpfully raised by an anonymous reviewer).

Finally, more cross-methodological and cross-disciplinary work must be performed in this area. For example, it may prove fruitful to probe whether there is greater cognitive effort or brain activity required to perceive the cluster based on salience or sonority poorness. This result, in particular, may also help further describe the nature of the relative salience and sonority values. The medium salience advantage may be related to cognitive effort.

## Figures and Tables

**Table 1:** The clusters developed for the study in addition to their scores along the salience and sonority dimensions.

Score Type	Score Value	Clusters	Count
Salience	Best	ft, kt, pt, tk, tl, vd, zd	7
Salience	Medium	dl, gd, tf	3
Salience	Worst	dn, dv, gn, lb, mn, rt	6
Sonority	Best	dl, dn, dv, gn, tf, tl	6
Sonority	Medium	gd, kt, mn, pt, tk	5
Sonority	Worst	ft, lb, rt, vd, zd	5

## Data Availability

The data presented in this study are available on reasonable request from the corresponding author due to copyright constraints on some of the visual stimuli. Auditory stimuli are available on OSF at https://osf.io/fws2b, accessed on 27 March 2026.

## Appendix A. Stimulus List

**Table A1:** Stimuli used for critical trials. Each novel creature ended with [ɹ], and each novel object ended with [n̩]. One competitor for each trial started with the first sound in the trial’s cluster, and the other competitor started with the second. Note that in each trial, one either the object or the creature had an epenthetic reduced vowel like [ə] breaking up the cluster.

Cluster	Item	Type
dl	dlɑpɚ	creature
dl	dlɑpn̩	object
dl	donkey	competitor
dl	lollipop	competitor
dn	dnɛzɚ	creature
dn	dnɛmn̩	object
dn	necklace	competitor
dn	dentist	competitor
dv	dvæɾɚ	object
dv	dvæbn̩	creature
dv	dancer	competitor
dv	vacuum	competitor
ft	ftɛlɚ	creature
ft	ftɛzn̩	object
ft	fairy	competitor
ft	tent	competitor
gd	gdʌpɚ	creature
gd	gdʌfn̩	object
gd	gum	competitor
gd	duckling	competitor
gn	gnuɾɚ	creature
gn	gnuln̩	object
gn	goose	competitor
gn	noodles	competitor
kt	ktʌgɚ	creature
kt	ktʌvn̩	object
kt	cupboard	competitor
kt	tunnel	competitor
lb	lbɛnɚ	creature
lb	lbɛkn̩	object
lb	leopard	competitor
lb	bench	competitor
mn	mnʌvɚ	creature
mn	mnʌpn̩	object
mn	mushrooms	competitor
mn	nuts	competitor
pt	ptɪsɚ	creature
pt	ptɪbn̩	object
pt	pickles	competitor
pt	tissues	competitor
rt	ɹtuɾɚ	creature
rt	ɹtufn̩	object
rt	rooster	competitor
rt	toothbrush	competitor
tf	tfɛʤ̑ɚ	creature
tf	tfɛɹn̩	object
tf	tennis	competitor
tf	feather	competitor
tk	tkæʤ̑ɚ	creature
tk	tkæsn̩	object
tk	taxi	competitor
tk	cabbage	competitor
tl	tlifɚ	creature
tl	tlinn̩	object
tl	teacher	competitor
tl	leaves	competitor
vd	vdɛgɚ	creature
vd	vdemn̩	object
vd	vet	competitor
vd	desk	competitor
zd	zdikɚ	creature
zd	zdiɹn̩	object
zd	zebra	competitor
zd	deer	competitor

## Appendix B. Statistical Results from GAMMs

**Table A2:** Table of coefficients for model comparing sonority and salience looks over time, including both parametric and smooth terms.

Parametric Term	Estimate	Std. Error	z Value	Pr (>|z|)
Intercept	−2.198	0.100	−21.940	<0.001
Looking at salience competitor	−1.278	0.016	−82.430	<0.001
Looking at sonority competitor	−1.344	0.016	−83.890	<0.001
Looking at epenthesis competitor	1.877	0.010	179.080	<0.001
**Smooth Term**	**edf**	**Ref.df**	**Chi.sq**	***p*** **-Value**
Time	0.006	0.006	0.001	0.973
Looks to epenthesis item over time	8.674	8.815	120.462	<0.001
Looks to salience competitor over time	7.691	8.283	72.052	<0.001
Looks to sonority competitor over time	8.599	8.847	138.398	<0.001
Looks to target over time	8.113	8.378	46.636	<0.001
By-event random smooths for time	1430.0	1566.0	16,420.228	<0.001

**Table A3:** Table of coefficients for salience model, including both parametric and smooth terms.

Parametric Term	Estimate	Std. Error	z Value	Pr (>|z|)
Intercept	−0.114	0.111	−1.024	0.306
Medium salience	0.533	0.185	2.876	0.004
Best salience	−0.157	0.127	−1.240	0.215
**Smooth Term**	**edf**	**Ref.df**	**Chi.sq**	***p*** **-Value**
Time	0.003	0.005	1.135	0.287
Time-by-worst salience	1.002	1.005	0.571	0.431
Time-by-medium salience	1.003	1.005	1.037	0.402
Time-by-best salience	2.920	3.705	17.920	0.001
Time-by-Subject random smooths	71.228	584.000	265.314	<0.001

**Table A4:** Table of coefficients for sonority model, including both parametric and smooth terms.

Parametric Term	Estimate	Std. Error	z Value	Pr (>|z|)
Intercept	−0.146	0.114	−1.281	0.200
Medium sonority	−0.216	0.143	−1.512	0.130
Best sonority	0.338	0.143	2.364	0.018
**Smooth Term**	**edf**	**Ref.df**	**Chi.sq**	***p*** **-Value**
Time	0.003	0.006	56.713	0.001
Time-by-worst sonority	1.003	1.005	2.362	0.109
Time-by-medium sonority	1.234	1.424	1.229	0.261
Time-by-best sonority	1.004	1.007	4.837	0.053
Time-by-Subject random smooths	73.902	584.0	414.871	<0.001
